# Associations between significant head injury and cognitive function, disability, and crime in adult men in prison in Scotland UK: a cross-sectional study

**DOI:** 10.3389/fpsyt.2025.1544211

**Published:** 2025-03-19

**Authors:** Tom M. McMillan, Hira Aslam, Abi McGinley, Vicky Walker, Sarah J. E. Barry

**Affiliations:** ^1^ School of Health and Wellbeing, University of Glasgow, Glasgow, United Kingdom; ^2^ Department of Psychiatry & Behavioural Neurosciences, McMaster University, Hamilton, ON, Canada; ^3^ Department of Mathematics and Statistics, University of Strathclyde, Glasgow, United Kingdom; ^4^ Department of Statistics, Frontier Science Scotland (Ltd), Kingussie, United Kingdom

**Keywords:** traumatic brain injury, head injury, prison, crime, disability, cognitive function

## Abstract

**Background:**

Although a history of head injury is common in prisoners, little is known about its impact in relation to disability and potential associations with other health problems. This is relevant to the development of effective management and interventions targeted towards health or reducing recidivism. This study investigates effects of significant head injury (SHI) on disability, cognitive function, and offending in adult male prisoners and considers relationships with common comorbidities.

**Methods:**

In this cross-sectional study, adult male prisoners in Scotland were recruited from Her Majesty’s Prisons Low Moss and Shotts. To be included, prisoners had to be men in adult custody, fluent in English, able to participate in assessment, provide informed consent, and not have a severe acute disorder of cognition or communication. History of head injury, cognition, disability, history of abuse, health, and problematic substance use were assessed by interview and questionnaire. Comparisons were made between prisoners with and without a history of SHI.

**Results:**

The sample of 286 was demographically representative of approximately 8,000 adult men in prison in Scotland. Severe head injury (SHI) was found in 245/286 (86%) and was repeated over periods of time in 151/245 (62%). Disability was associated with SHI in 85/245 (35%) and was significantly associated with problematic drug or alcohol use, clinical anxiety, and clinical depression. Significant associations between SHI and cognitive test outcomes were not found. Prisoners with SHI had more arrests, charges, and convictions and at younger ages, and were at greater risk of involvement in violent and property offences.

**Conclusions:**

A history of repeated SHI is very common in adult men in prison and is associated with a greater risk of crime including violence. Disability after SHI often affects social relationships and is associated with multiple health problems. There is a need for policy and interventions to account for the “weave” of multiple health needs of people in prison, which includes history of SHI.

## Introduction

The worldwide prison population continues to grow and, with this, an economic and social burden, with the UK, including Scotland, having amongst the highest prison population in Europe (1) (World Prison). Linked to this, there is a debate about sentence length and early release as a pragmatic solution to overcrowded prisons, with concern about desistance and impact on crime and reconviction rates (2–5). Clearly, there is a need to understand the factors associated with risk of criminal behaviour and how they interact in order to reduce the risk of offending.

It is understood that the health of people in prison is poorer than in the general public and that this can be associated with offending (6, 7). A significant body of research has looked at history of head injury in people involved in the criminal justice system as one such health factor (8). Meta-analyses suggest that the prevalence of head injury is high at approximately 45%–60% (9–11) although a wide range from almost 0 to 100% has been reported (12). Birth cohort and population studies that link databases on health and criminal conviction in adult life support the view that the prevalence of head injury is high in people involved in crime and that offending cannot be easily explained by environmental factors alone (13, 14). Theories of criminal behaviour include cognitive and emotional factors such as impairment in judgement, flexibility of thinking, and self-control (15, 16), and these are common sequelae of head injury (17). However, these factors can also be associated with other characteristic features of a prison population such as a history of deprivation, multiple health morbidity, and problematic substance use. There is a need therefore to consider this “weave” of health-related problems commonly found in prisoners and the role of head injury in this. What is also unclear is the extent to which adult men in prison with a history of head injury have been disabled by it and in what ways (12). Knowledge about relationships between head injury and other health morbidities and of disability arising from head injury is important when developing and targeting support and intervention. There is also a need to understand the extent to which head injury remains a factor that is associated with offending if taking into account other common health problems in prisoners including mental health, substance use, and trauma. We reported these relationships and disability in women and in young men in prison in Scotland (18, 19), and now we do so in adult men.

We use the term “head injury” here rather than traumatic brain injury. Head injury indicates that there has been trauma to the head, and usually, there is little doubt about this. “Traumatic brain injury,” however, is a consequence of head injury that makes an assumption about brain damage and may imply that this is a significant insult, whereas this is not the case for many where the injury was mild or any damage extracranial. This distinction is particularly important for people involved in the criminal justice system because they may not attend hospital and the head injury was not assessed (20). We use the term offender in this study for clarity and in accordance with current usage in the criminal justice and forensic mental health systems, and it is not intended to be pejorative.

The aim of the present study is to investigate the prevalence of significant head injury, other health problems, and relationships with cognitive function, associated disability, and characteristics of offending in a representative sample of the adult male Scottish prison population.

## Methods

The study took place in two prisons in Scotland, HMPs Low Moss and Shotts. These prisons house adult men aged 21 years and over, including those with short-term (<4years), long-term (>4 years), life, and extended life sentences. Low Moss also takes prisoners on remand. HMP Low Moss and Shotts have capacities of approximately 850 and 550 prisoners, respectively. Healthcare is provided to these prisons by the National Health Service.

The study only included adult male participants, while our other studies have focused on adult women (18) and young men (19). There are approximately 8,000 people in prisons in Scotland, of which approximately 90% are adult men (21). Prisoners were recruited between 2 February 2017 and 30 August 2019. To be included, they had to be fluent in English, not have a severe acute disorder of cognition or communication, and give written informed consent. Participants were recruited through word of mouth and posters placed in prison halls. To avoid bias towards recruiting participants with head injury, the project was advertised on posters as a study on prisoner health. Potential prisoner participants then met with a researcher (HA, AMcG, or VW) to find out more about the study and provide consent. It was made clear to potential participants that they could withdraw from the study at any time and did not need to give an explanation. It was also made clear to them that taking part in the study would not affect any treatment that they may be undergoing or their custodial sentence. If a researcher became aware of a significant health issue for a participant, this information was passed to the prison healthcare staff with the participant’s consent. If a researcher became concerned about a participant’s risk to self or others, this information would be passed on to the relevant prison officer to enable them to use relevant Scottish Prison Service (SPS) risk management policies and procedures, and signpost relevant agencies, and, if deemed necessary, this action would be taken without the participant’s consent.

### Procedure

Assessments took 1–2 h with breaks if needed and were carried out by final year doctorate in clinical psychology trainees (AMcG, VW) or by an experienced research worker (HA). Details of assessor training are given in the **Supplementary Material**, If the participant was fatigued or seemed unwell, as indicated by self-report or by observation of the assessor, the assessment was completed in a second session.

### Measures

Participants completed a background information questionnaire that included questions about age, ethnicity, schooling, occupation, history of drug and alcohol use, offending history, and length of hospital stay with head injury. Deprivation quintiles were derived from postcodes using the Scottish Index of Multiple Deprivation (22) and combined into high deprivation (quintiles 1–2) vs. low deprivation (quintiles 3–5) due to small numbers. SIMD 2020 ranks deprivation in Scotland across 6,976 small geographical areas and considers income, employment, education, health, access to services, crime, and housing.

#### Head injury

This was assessed using one of two structured interviews. The Ohio State University Traumatic Brain Injury Identification Method (OSU-TBI) is a structured interview for the assessment of history of head injury. It is valid for prison samples (23) (Bogner) and practical to use in prisons in Scotland (18, 19). The OSU-TBI records information on cause and severity of single-event and multiple head injury. The Brain Injury Screening Index (BISI) is a brief assessment of head injury by self-report for use in prison populations (24). These tools have been shown to have similar sensitivity and specificity with regard to identification of head injury (25).

#### Other CNS damage

Participants were asked a series of questions based on those provided in association with the OSU-TBI and BISI. In addition, any other references to other CNS conditions during the interview were noted if a participant indicated that they had lost consciousness for a cause other than head injury (e.g., anoxia) and if indicating disability from a cause other than HI.

#### Cognitive function

Three tests were given to assess processing speed, attention, and visual scanning (Symbol Digit Modalities Test) (26), auditory verbal list learning (27), processing speed, and mental flexibility (Trail Making Test) (28). The Word Memory Test assessed effort during cognitive test performance with a score below 34 at delayed recall suggesting poor effort (29).

#### Disability

The Glasgow Outcome at Discharge Scale (GODS) is a standardised, structured assessment of disability developed from the Glasgow Outcome Scale-Extended for use when individuals are not in the community (30). Disability was rated as attributable to head injury or from any cause. The wording (but not structure) was altered to suit a prison context (18).

#### Psychological distress

The Hospital Anxiety and Depression Scale (HADS) was used to assess anxiety and depression by self-report with scores above 10 indicating moderate–severe caseness (31).

#### Trauma

Due to time constraints, questionnaires pertaining to trauma history and PTSD were given to a random sample of the study participants from one prison. The Traumatic Life Events Questionnaire (TLEQ) (32) and the PTSD Checklist for the DSM-5 (PCL-5) (33) were used to assess lifetime exposure to traumatic events and PTSD, respectively. A score above 32 and fulfilment of criteria for intrusion, avoidance, and hypervigilance on the PCL-5 suggests PTSD (34). The Adverse Childhood Experiences questionnaire (ACE) (35) assesses exposure to adverse life experiences before age 18; a score of >3 was used to indicate risk of significant mental health problems (36). These measures have been used successfully with Scottish prisoners (18, 19).

### Definition of groups

Participants were grouped as having significant head injury (SHI) if reporting a mild head injury with loss of consciousness for <30 min or moderate-to-severe head injury with loss of consciousness for at least 30 min, or head injury without loss of consciousness on more than two occasions with acute effects such as dizziness or feeling dazed. Offenders were classified without SHI if reporting no history of head injury or head injury without loss of consciousness on fewer than three occasions or without loss of consciousness or acute effects (NoSHI group) (18, 37).

### Data analysis

Analyses were performed in R (v-4.2.2). Continuous variables were summarised using mean and standard deviation (SD) or median and interquartile range (IQR), depending on the variable distribution. Categorical variables were summarised using counts and percentages. Two-sided t-tests or Mann–Whitney tests were used as appropriate to assess differences between the head injury groups in participant characteristics measured as continuous variables, while Fisher’s exact test or the chi-squared test was used for categorical variables. Age, years of education, and delayed Word Memory Test score were used to adjust cognitive test scores, and an overall summary adjusted z-score was subsequently calculated. Disability outcomes from the GODS assessment were aggregated into severe disability, moderate disability, or good recovery/no disability (and no SHI, for HI-attributed analysis). Number of arrests and charges were truncated to 200 and number of convictions to 100 due to unrealistically high self-report numbers in a few cases.

A linear regression model was fitted to the continuous outcome (cognitive impairment z-score), logistic regression models to the binary outcomes (disability: moderate/severe vs. good recovery/no disability) and violent offending (any violent offence vs no violent offences), and quasi-Poisson models to the count outcomes (number of convictions and longest sentence), allowing for overdispersion. Univariable models assessed the unadjusted differences between SHI groups, while multivariable models adjusted for four relevant contributing factors (problematic drug or alcohol use; CNS diagnosis at any age, current clinical depression or current clinical anxiety, both as defined by the HADS). The model for head-injury-attributed disability included only those in the SHI group and assessed the impact of the four contributing factors.

Model estimates of the group differences are reported as mean difference (linear model), odds ratio (logistic model), or rate ratio (quasi-Poisson model), with corresponding 95% confidence intervals.

Pearson correlation coefficients were used to assess collinearity between explanatory variables, with the highest correlation of 0.36 being between current depression and current anxiety and all others being below 0.2. We considered these low enough to justify inclusion of these explanatory variables in the models.

### Ethics approvals

Permission was obtained from the West of Scotland NHS Research Ethics Committee (16/WS/0216) and from the Scottish Prison Service Research Ethics Committee (no reference; date 26 August 2016). Written consent was obtained from all participants.

## Results

The average age of the sample of 286 adult men was 37 years (SD, 10; range, 21–71). Almost all (272/286; 95%) self-described as ethnically white. Five self-described as Asian, four as mixed-race or multi-race, three as traveller, and one as “other.” More than half were from the most socially deprived quintile (**Table 1**). The study sample is demographically representative of the Scottish prison population. Scottish prison population statistics for 2019–2020 (21) reveals very similar demographics with an average age of 36 years, 95% self-identifying as ethnically white, and 53% from the most socially deprived quintile.

**Table 1:** Baseline characteristics.

Variable	Statistic	All (N = 286)	SHI (N = 245)	NoSHI (N = 41)	p-value
Age (years)	N_obs_ (N_miss_)	286 (0)	245 (0)	41 (0)	
	Mean (SD)	37 (10)	36 (11)	37 (10)	
	Range	(21, 71)	(21, 71)	(22, 67)	0.805
Ethnicity	N_obs_ (N_miss_)	285 (1)	244 (1)	41 (0)	
White	N (%)	272 (95%)	236 (97%)	36 (88%)	
Non-white	N (%)	13 (5%)	8 (3%)	5 (12%)	0.026
SIMD quintile high/low	N_obs_ (N_miss_)	234 (52)	201 (44)	33 (8)	
1:2 (high deprivation)	N (%)	187 (80%)	160 (80%)	27 (82%)	
3:5 (lower deprivation)	N (%)	47 (20%)	41 (20%)	6 (18%)	1.000
Years of education	N_obs_ (N_miss_)	284 (2)	243 (2)	41 (0)	
	Median (IQR)	11 (10,12)	11 (10,12)	11 (10, 12)	
	Range	(0, 20)	(0, 20)	(8, 20)	0.931

The median time in education was 11 years (IQR, 10,12). However, almost half had received 1:1 support in school or special schooling (139/284; 48%), and a further three reported not attending school. Most had truanted (247/282; 88%) with 178/282 (63%) reporting having done so weekly and schooling being further disrupted by suspension or exclusion in more than three quarters (216/281; 77%). More than a quarter had been unemployed prior to incarceration (78/285; 27%). Differences between SHI (n=245) and NoSHI groups (n=41) were not statistically significant for age, socioeconomic deprivation, years of education, school attendance or employment (**Supplementary Material**). The exception was ethnicity, where more men self-described as non-white in the No-SHI group, although the numbers are very small (**Table 1**).

### Occurrence of head injury

Most participants reported a history of SHI (245/286; 86%). The “worst” head injury was severe (LoC > 24 h) in 28/244 (11%) and was moderate (LoC, 30 min to 24 h) in 57/244 (23%). LoC was reported but was of unknown duration in 3/244 (1%). For the remainder, the “worst” head injury was mild, with LoC for <30 min in 132/244 (54%) and mild without LoC in 24/244 (10%). The SHI group comprised of these 245 participants plus one with LoC of unknown duration. Few had a history of a single SHI with LoC without repeat SHI (20/244; 8%). Of those that did, 13 had mild, four moderate, and three severe SHI. All participants in the SHI group reported having symptoms after head injury. Almost two-thirds of the SHI group reported one or more periods of time when they sustained repeated head injury (150/244; 61%). In the SHI group, the median age at the first head injury was 10 years (IQR, 6,15), both in the group as a whole and in those with LoC. The majority had one or more head injuries in childhood; 20% had a head injury before age 6, 42% before age 10, and 84% before the age of 18. Many in the SHI group reported not ever attending hospital after a head injury (90/232; 39%). Some, however, reported having been in hospital after a head injury for considerable periods of time; for example, 48/232 (21%) for more than a week and 20/232 (9%) for more than a month (**Supplementary Material**). The NoSHI group comprised 41 participants. Of these, 7 (17%) had no history of head injury, 23 (56%) had one or two head injuries without LoC, and 11 (27%) had two or more HI without LoC or acute symptoms. The most common lifetime cause of SHI was assault in 210/245 (86%), and most often, this was in the context of gang or “scheme” fighting (149/210; 71%). Other causes were falls in 86/245 (35%), a vehicle injury in 85/245 (35%), sporting activities that were most often boxing, football, and cycling in 80/245 (33%), and other causes (60/245; 24%), which were most often accidents. Relatively few in the SHI group reported head injury resulting from parental or other family violence (21/245; 9%) including partner violence (7/245; 3%). These figures are based on the total number of head injuries or episodes reported in the SHI group, with most individuals having head injuries from more than one cause (179/245; 73%). In the SHI group, age at first HI was not significantly associated with SHI-related disability (OR, 0.99; 95% CI, 0.95–1.02), overall cognitive function (difference in means, −0.01; 95% CI, −0.02–0.01), or violent offending (OR, 0.97; 95% CI, 0.93–1.01).

### Occurrence of other central nervous system diagnoses

A CNS diagnosis (other than HI) at any age was self-reported by 91/285 (32%), of which 83/244 (34%) were in the SHI group and 8/41 (20%) in the NoSHI group (p = 0.072). A diagnosis of ADHD was self-reported by 36/286 (13%) with all but one being in the SHI group (35/245; 14%). Epilepsy/seizures with unclear cause were reported in 21/286 (7%) and alcohol/drug related seizures in 23/286 (8%). For other conditions, the numbers were small, including learning disability (12/286; 4%), cerebral anoxia (9/286; 3%), autistic spectrum disorder (5/286; 2%), stroke (4/286; 1%), childhood meningitis (3/286; 1%), alcohol-related brain damage (2/286; 1%), and Parkinson’s disease (1/286; 1%) (**Supplementary Material**).

### Mental health and substance use

Two-thirds of participants self-reported having serious mental health problems (193/286; 67%), with anxiety (134/286; 47%) and depression (130/286; 46%) being most common. Difficulties resulting from anger or temper outbursts were self-reported in 88/286 (31%). Other mental problems by self-report were PTSD (23/286; 8%), personality disorder (21/286; 7%), and psychosis (13/286; 5%). Overall mental health problems were more often self-reported in the SHI group (171/245; 70%) than in the NoSHI group (22/41; 54%; p=0.048).

On the HADS, scores for anxiety and depression were both significantly higher in the SHI than in the NoSHI group (**Table 2**). Clinical anxiety was more common in the SHI group (108/241; 45%) than in the NoSHI group (8/41; 20%; p=0.004) as was clinical depression (SHI 50/242; 21%; NoSHI 1/41, 2%, p=0.01; **Table 2**). A self-reported history of problematic drug (210/284; 74%) or alcohol (146/284; 51%) use was common. Most reported problems with either drugs or alcohol (234/284; 82%), with 122/284 (43%) reporting problems with both. Differences between groups were non-significant.

**Table 2:** Mental health.

Variable	Statistic	All (N= 286)	SHI (N= 245)	NoSHI (N= 41)	p-value
HADS depression score	N_obs_ (N_miss_)	283 (3)	242 (3)	41 (0)	
	Median (IQR)	6 (3, 9)	7 (3, 9)	3 (2, 5)	
	Range	(0, 21)	(0, 21)	(0, 14)	<0.001
Clinical depression	N_obs_ (N_miss_)	283 (3)	242 (3)	41 (0)	
(HADS depression>10)	N (%)	51 (18%)	50 (21%)	1 (2%)	0.010
HADS anxiety score	N_obs_ (N_miss_)	283 (3)	242 (3)	41 (0)	
	Median (IQR)	9 (5, 13)	9 (6, 14)	5 (3, 10)	
	Range	(0, 30)	(0, 30)	(1, 14)	<0.001
Clinical anxiety	N_obs_ (N_miss_)	282 (4)	241 (4)	41 (0)	
(HADS anxiety>10)	N (%)	116 (41%)	108 (45%)	8 (20%)	0.004
PTSD diagnosis (PCL-5)	N_obs_ (N_miss_)	58 (228)	48 (197)	10 (31)	
	N (%)	28 (48%)	24 (50%)	4 (40%)	0.0732
PCL-5 score	N_obs_ (N_miss_)	58 (228)	48 (197)	10 (31)	
	Mean (SD)	36 (18)	37 (18)	33 (17)	
	Range	(0, 80)	(0, 80)	(10, 57)	0.546

### Physical or sexual abuse and trauma

The sub-group of 58/286 that was assessed on trauma measures did not differ significantly from those in the overall sample in age at assessment, ethnicity, high socioeconomic deprivation, or employment. Those who were assessed on trauma measures were on average enrolled for 1 year less at school [10 years (IQR, 8–12) assessed vs. 11 years (IQR, 10–12) not assessed; p=0.009) and were more likely to have received special schooling [38/58 (66%) assessed vs. 101/226 (45%) not assessed; p=0.030] (**Supplementary Material**). The proportion of the sub-group assessed on measures of trauma and PTSD that had SHI (48/58; 83%) was similar to that in the total sample (86%), with differences in the proportions participating from each group in the overall sample not statistically significant (p=0.619; **Supplementary Material**).

Almost all participants who completed the TLEQ reported a history of abuse (52/57; 91%), including abuse during childhood in two-thirds (37/57; 65%). The average score on the TLEQ was 9 (SD 3; range 3,15), with no difference between groups. On the ACE, adverse childhood experiences were reported in almost all participants (56/58; 97%). The average score on the ACE was 6 (SD 3), with almost all participants in (56/58 (97%) having scores above 3 that indicate a significant risk of trauma-related health difficulties (Hughes 17). On the PCL-5, scores were above the clinical cutoff of 33 in 34/58 (59%) and almost half-fulfilled diagnostic criteria for PTSD (28/58; 48%). There was no evidence of group differences by SHI on the ACE or PCL-5 (**Supplementary Material**).

### Disability

In the SHI group, disability on the GODS that was attributed to SHI was found in 85/245 (34%). Disability was commonly associated with adverse effects on social relationships arising from mental health problems (77/86; 90%). More specifically, these were self-reported as depression (62/85; 73%), anxiety (58/85; 68%), and anger control (45/85; 53%). Almost three quarters of those disabled by SHI reported more than one associated mental health complaint (60/85; 71%). SHI was the only cause of disability in 30/245 (12%) in the SHI group. In a multivariable logistic regression model, participants with self-reported problematic drug or alcohol use (OR, 3.64; 95% CI, 1.34, 9.89), current clinical depression (OR, 2.07; 95% CI,1.03, 4.16), or current clinical anxiety (OR, 1.92; 95% CI, 1.06, 3.46) were significantly more likely to have SHI-attributed disability (vs. good recovery or no SHI-attributed disability). There was no evidence of an association between SHI-attributed disability and history of other CNS disorders (OR, 1.45; 95% CI, 0.81, 2.61) (**Supplementary Figures**).

Disability from any cause (SHI and/or other causes) was found in 164/286 (57%) with group differences in the presence or absence of disability from any cause non-significant overall (p=0.087) or by disability categories (p=0.151) (**Supplementary Materials**). Those reporting disability from any cause most commonly reported that it was related to social relationships arising from mental health problems (147/164; 90%), particularly anxiety (111/164; 68%) and low mood (108/164; 66%). Disability arising from anger control was also common (77/164; 47%). Personality disorder was reported in 17/164 (10%) and psychotic symptoms, often in the context of effects of drug use in 9/164 (6%). Physical (non-CNS) causes of disability were rarely reported (9/164; 6%) and were from a variety of conditions, with 3/164 (2%) reporting reduced mobility as a cause. Most reported more than one mental health complaint (104/164; 63%) (**Table 3**, **Supplementary Materials**).

**Table 3:** Disability on the glasgow outcome at discharge scale .

Variable	Statistic	All (N = 286)	SHI (N = 245)	NoSHI (N = 41)	p-value
Disability from any cause	N_obs_ (N_miss_)	286 (0)	245 (0)	41 (0)	
Good recovery/no disability history	N (%)	122 (43%)	99 (40%)	23 (56%)	
Moderate disability	N (%)	127 (44%)	114 (47%)	13 (32%)	
Severe disability	N (%)	37 (13%)	32 (13%)	5 (12%)	0.151
Disability from SHI	N_obs_ (N_miss_)	286 (0)	245 (0)	41 (0)	
Good recovery	N (%)	192 (67%)	160 (65%)	–	
Moderate disability	N (%)	77 (27%)	77 (31%)	–	
Severe disability	N (%)	8 (3%)	8 (3%)	–	–

In a univariable model, the association between SHI and disability from any cause approached statistical significance (OR, 1.88; 95% CI, 0.97, 3.67). However, when potential risk factors for disability from any cause were included in a multivariable model, the effect estimate for SHI reduced considerably in magnitude, and there was no longer any evidence of an association (OR, 1.15; 95% CI, 0.54, 2.43). Current clinical anxiety (OR, 4.67; 95% CI, 2.54, 8.58), self-reported drug or alcohol use (OR, 3.62; 95% CI, 1.72, 7.61), and other CNS diagnosis (OR, 2.52; 95% CI, 1.37, 4.66) showed strong associations with any cause disability, while current clinical depression did not (OR, 1.47; 95% CI, 0.63, 3.43) (**Supplementary Figures**).

### Cognitive function

Cognitive tests revealed little difference between SHI and NoSHI groups, either as raw scores or after adjustment for age, years of education, and delayed Word Memory Test score (**Supplementary Material**). Correspondingly, the overall cognitive z-score showed little difference between groups overall (SHI mean, −0.019; SD, 1.025; NoSHI mean, 0.112; SD, 0.839; p=0.441). Findings were similar if only including those who were above the cutoff on the delayed Word Memory Test (N=203; SHI mean, 0.012; SD, 0.999; NoSHI mean, 0.147; SD, 0.879; p=0.476) or after multivariable adjustment for relevant contributing factors (difference in means, −0.04; 95% CI, −0.37–0.30) (**Supplementary Figures**). Risk factors were not associated with cognitive scores in multivariate analysis (**Supplementary Material**).

Most participants, 203/284 (71%), scored above the cutoff score of 33 on delayed recall of the Word Memory Test, suggesting reasonable effort on cognitive tests. Differences in the proportion above the cutoff of 33 for the Word Memory Test (SHI, 173/243, 71%; NoSHI, 32/41, 78%) were not significant by group (p=0.473) (**Supplementary Material**).

### Offending history

The SHI group had more arrests, charges, and convictions than the NoSHI group. On average, the SHI group was 1 year younger at first arrest [SHI 15 years (IQR, 13–16) vs. NoSHI 16 years (IQR 14–21); p<0.001], 4 years younger at first charge [SHI 16 years (IQR, 14–17) vs. NoSHI 20 years (IQR, 16–25); p<0.001], and 5 years younger at first conviction [SHI 16 years (IQR, 15–21) vs. NoSHI 21 years (IQR, 18–34); p=0.005]. The longest length of sentence did not differ significantly between groups (**Table 4**).

**Table 4:** Offending history.

Variable	Statistic	All (N = 286)	SHI (N = 245)	NoSHI (N = 41)	p-value
Number of arrests (truncated to 200)	N_obs_ (N_miss_)	280 (6)	240 (5)	40 (1)	
	Median (IQR)	20 (9, 50)	20 (10, 50)	10 (5, 40)	
	Range	(1, 200)	(1, 200)	(1, 200)	0.010
Number of charges (truncated to 200)	N_obs_ (N_miss_)	279 (7)	240 (5)	39 (2)	
	Median (IQR)	18 (7, 40)	20 (8, 40)	8 (3, 35)	
	Range	(1, 200)	(1, 200)	(1, 150)	0.011
Number of convictions (truncated to 100)	N_obs_ (N_miss_)	282 (4)	242 (3)	40 (1)	
	Median (IQR)	10 (3, 25)	10 (4, 30)	6 (2, 15)	
	Range	(1, 100)	(1, 100)	(1, 57)	0.009
Longest length sentence (months)	N_obs_ (N_miss_)	274 (12)	235 (10)	39 (2)	
	Median (IQR)	27 (13, 60)	25 (12, 60)	36 (18, 68)	
	Range	(0, 264)	(0, 240)	(4, 264)	0.183
Violent offences	N_obs_ (N_miss_)	284 (2)	243 (2)	41 (0)	
Yes	N (%)	245 (86%)	216 (89%)	29 (71%)	0.005
Sexual offences	N_obs_ (N_miss_)	284 (2)	243 (2)	41 (0)	
Yes	N (%)	9 (3%)	8 (3%)	1 (2%)	1.000
Property offences	N_obs_ (N_miss_)	284 (2)	243 (2)	41 (0)	
Yes	N (%)	156 (55%)	141 (58%)	15 (37%)	0.017
Other offences	N_obs_ (N_miss_)	284 (2)	243 (2)	41 (0)	
Yes	N (%)	239 (84%)	202 (83%)	37 (90%)	0.355
Age at first arrest (years)	N_obs_ (N_miss_)	272 (14)	232 (13)	40 (1)	
	Median (IQR)	15 (13, 17)	15 (13, 16)	16 (14, 21)	
	Range	(8, 70)	(8, 70)	(8, 64)	<0.001
Age at first charge (years)	N_obs_ (N_miss_)	223 (63)	193 (52)	30 (11)	
	Median (IQR)	16 (14, 18)	16 (14, 17)	20 (16, 25)	
	Range	(8, 70)	(8, 70)	(9, 64)	<0.001
Age at first conviction (years)	N_obs_ (N_miss_)	135 (151)	118 (127)	17 (24)	
	Median (IQR)	16 (15, 22)	16 (15, 21)	21 (18, 34)	
	Range	(8, 70)	(8, 70)	(9, 64)	0.005

Multivariable analysis indicated that problematic alcohol or drug use was associated with having more convictions (RR, 3.82; 95% CI, 2.14–6.82), as was clinical depression (RR, 1.39; 95% CI, 1.03–1.88), while SHI, clinical anxiety, and other CNS diagnosis showed no evidence of an association with number of convictions. There was no evidence that SHI, problematic alcohol or drug misuse, current clinical anxiety or depression, or other CNS diagnosis were associated with longest length of sentence (**Supplementary Material**).

A history of violent offences was very common and significantly more so in the SHI group than in the NoSHI group, as were property offences (**Table 4**). Multivariable analysis indicated that SHI (OR, 2.94; 95% CI, 1.25, 6.89) and a self-report of problematic drug or alcohol use (OR, 4.21; 95% CI, 1.91, 9.24) were risk factors for violent offending, while clinical anxiety had a marginal association (OR, 2.44; 95% CI, 0.99, 6.02), and clinical depression (OR, 0.78; 95% CI, 0.23, 2.63), and other CNS diagnosis (OR, 1.28; 95% CI, 0.53, 3.07) showed no evidence of an association with violent offending (**Supplementary Figures**).

## Discussion

In this cross-sectional study, SHI was found in more than four out of five adult male prisoners in Scotland, and this is higher than estimates of 45%–60% reported in meta-analyses (9–11). There are several factors to note here. Studies included in the meta-analyses report a wide range of prevalence (3%–100%). Unlike the present study, most studies in the meta-analyses do not report whether their sample is representative of their population, several did not use validated tools to assess head injury, and some meta-analyses included studies with small samples (11, 12). These are important considerations in the design of future studies and need to be borne in mind when planning services. Nevertheless, there is a weight of evidence to indicate that a history of head injury is highly prevalent in people in prison. What is more, almost two-thirds of our sample had a history of repeated head injury over long periods of time that was largely of mild severity. This pattern is very similar to that reported in our studies on adult women and young men in prison in Scotland (18, 19). A history of repeat mild head injury in >50% of prisoners has been widely reported by others (38–40) and can result in cumulative brain damage (23, 41, 42). This is of particular concern in people involved in the Criminal Justice System (CJS), given that they often do not attend hospital and tend not to be aware of potential long-term effects of head injury, and this history may be missed in screening if not using appropriate validated measures (20, 23, 43).

The impact of head injury on the day-to-day life of people in the CJS has received less focus but is clearly important in understanding relationships with criminal behaviour and in developing and targeting support and interventions. Very few studies in criminal justice have assessed disability associated with head injury (12, 18, 19, 44, 45) (**Supplementary Material**). In the present study, a third of adult men were disabled by SHI. This is similar to findings in adult women in Scotland, whereas disability associated with SHI occurred in only one in eight young men in prison (18, 19). Similarly and of note, disability was largely associated with difficulties with social relationships that were linked to mental health problems and poor temper control. Clinical anxiety, depression, and substance use problems were risk factors for disability from SHI and consistent with the disability-related difficulties with social relationships that were found. Health complaints are common in prisoners (7), and our sample was no exception. Almost all reported a history of abuse and trauma, and two-thirds had current mental health problems, which were most often anxiety, depression, and PTSD. The absence of a significant effect of SHI on the occurrence of PTSD in the adult men in this study might reflect the very high prevalence of abuse in the overall sample. However, findings are different in women in prison where there is a higher risk of PTSD in those with SHI (18). The difference might be explained by cause of SHI. Whereas fighting/assault was the most common cause of head injury in men, violent abuse by a partner was most common in women (18). Four in five men reported substance use problems, and a history of CNS disorders other than head injury was reported by a third. Together with mental health, these multiple complaints attest to a “weave” of health-related problems in which SHI is a strand for many, but not all. About a quarter of the SHI group were disabled from other causes but not disabled by head injury. This emphasises the importance of not assuming that disability in someone with a history of SHI is caused by head injury and of using a structured interview tool that allows exploration of responses about disability including cause (30). Hence, there is a need to consider the lifetime history of SHI, including in interventions that focus on mental health or substance use, and moreover, our findings support the case for holistic approaches that take account of health multimorbidity in addition to environmental factors and the personal circumstances of the individual.

Cognitive test scores did not discriminate between adult male prisoners with and without SHI. This is consistent with our studies on adult women and young men in Scottish prisons (18, 19) and with most (46–51) but not all (24) other studies. The absence of a group effect may reflect generally poorer cognitive function in prisoners than in the general population that could arise from the history of deprivation, disadvantage, and poorer health that is commonly found (16, 52, 53). Indeed, a history of this kind was found here and in our other studies, with social deprivation, limited opportunities in education, and multiple health problems being characteristic of the overall samples and scores on cognitive tests lower than expected from test norms (18, 19) (**Supplementary Material**).

SHI was associated with involvement in the CJS in a number of ways. Those with SHI became involved with the CJS at an earlier age and had more convictions, although the latter became attenuated after adjusting for relevant contributing factors such as problematic alcohol or drug use. There was also a higher risk of violent (and property) crime in the SHI group. Other studies report a similar association between head injury and violent offending (13, 14, 18). This is not surprising given that traumatic brain damage is known to reduce self-control (17), which in turn is considered to be a key risk factor for antisocial and criminal behaviour (15). In addition to SHI, violent crime was associated with problematic drug or alcohol use in our study, and this may reflect aggressive interactions when obtaining, using, or selling drugs, including the potential for head injury to act synergistically with drugs to reduce self-control. This should be considered when planning remedial and support programmes for people with substance use problems.

Adult men in prison with SHI differ from the general population with a history of head injury, and this needs to be reflected in planning services and support. In addition to the higher prevalence in prisoners (54), people involved in the CJS are characterised by head injury that most often results from assault and fighting, by having repeat head injury over long time periods and with most sustaining their first head injury in childhood. The general population with head injury differs in the UK, a fall is the most common cause, and a history of single incident accidental head injury is more typical (55, 56). Prisoners returning to the community are likely to return to the same environment, with the same elevated risk of further head injury and consequent cumulative brain damage. Their lack of understanding about the impact of head injury on their lives is likely to continue, especially given the tendency not to attend hospital, where they might be given information and be offered advice and links to head injury services. There is a strong need, therefore, to educate prisoners about head injury. Brief group-based education in prison about the causes and effects of head injury can improve knowledge and can incorporate simple strategies that may reduce the risk of violence (43). Education might help with management problems in prison, where recorded incidents including violence are more common in prisoners with a head injury history (19, 46, 57, 58). Given this, there is also a need to train staff in the CJS about head injury and its management. Education and training are relatively low in cost and potentially scalable for prison populations and may have potential to reduce management problems and have impact on recidivism rates, which are generally high and greater in prisoners with a history of head injury (2, 20, 39, 58–62). Arising from this picture is a need for support during transition and in the community. The evidence based on interventions and support for people in transition from prison to community is sparse, and given the development of our understanding of relationships between head injury and crime, there is now a need to address this (63).

The study has a number of strengths including the representative nature of the sample and the use of validated measures. Attempts were made to minimise sources of error including use of more than one assessor (**Supplementary Material**); nevertheless, there are a number of limitations. Data were largely obtained from retrospective self-report, and this could introduce error through inaccuracies in memory. The use of validated measures that allow prompting is likely to reduce error from self-report, and it should be noted that history of head injury cannot be obtained from medical records given that people involved in the CJS often do not attend hospital. Two tools were used to assess lifetime history of head injury, and they might produce differences. However, there were no significant differences in the prevalence of SHI detected by these tools, and both have been used repeatedly in studies in the CJS. Data on trauma were only obtained in a proportion of the sample. Although those with trauma data were similar demographically to the overall sample, caution is required in interpreting this data. It was not possible to adjust the models for trauma variables due to the trauma data only being collected on a proportion of the sample. The prevalence of SHI and some contributing factors was high, making statistically modelling challenging and was associated with some wide confidence intervals.

In conclusion, there is a high prevalence of SHI that is often repeated in adult male prisoners and is associated with multiple health complaints, life challenges, disability that affects social relationships, and a greater risk of crime including violence. Despite a growing and robust evidence base that supports associations between SHI and involvement with criminal justice, there has been a limited focus on interventions and support (63). There is therefore a need for a focus on a policy-driven approach, taking a holistic view of the “weave” of multiple health morbidity and incorporates interventions that can reduce the impact on behaviour and risk of future head injury and that is linked to support in prison and transition into the community.

## Data Availability

The datasets presented in this article are not readily available because there is no permission from the ethics board for data to be made available. Requests to access the datasets should be directed to emmajane.gault@glasgow.ac.uk.
