# Supplementary material for: Associations between significant head injury and cognitive function, disability, and crime in adult men in prison in Scotland UK: a cross-sectional study
Source: Front Psychiatry. 2025 Mar 19;16:1544211. doi: 10.3389/fpsyt.2025.1544211 (PMC11961919; doi:10.3389/fpsyt.2025.1544211)
Supplement: Supplementary file 1 [file SupplementaryFile1.docx]

V3

**Supplement**

**Literature Search**

A literature search was carried out to update those previously reported^1,2^. The purpose was to determine whether there is further published evidence on disability in offenders with head injury. The following databases were searched: PsycINFO (EBSCO), CINAHL (EBSCO) and Medline (OVID) from 1 January 2021 to 25 November 2024. The text word search was:

(traumatic brain injury or head injury, or brain injury or tbi) AND

(crim* or offend* or prison* or inmate) AND

(disable* or disabil*)

The search revealed 41 journal articles. Disability after head injury was assessed in only two:

De Mora H, McFarlane J, McMillan TM (2024). Prevalence of head injury and associated disability in individuals undergoing pre-sentencing assessment by Criminal Justice Social Work. Forensic Science International: Mind and Law 5,100029. <https://doi.org/10.1016/j.fsiml.2024.100129>

McMillan TM, McVean J, Aslam H, Barry SJE (2023). Associations between significant head injury in male juveniles in prison in Scotland UK and cognitive function, disability and crime: A cross sectional study. PLOS ONE 18(7): e0287312 <https://doi.org/10.1371/journal.pone.0287312>

^1.^McGinley A, McMillan TM (2019). The Prevalence, Characteristics and Impact of Head Injury in Female Prisoners: A PRISMA Systematic Review. Brain Inj: 33:1581-91

^2.^McMillan TM, Aslam H, Crowe E, Seddon E, Barry SJE (2021). Associations between significant head injury and persisting disability and violent crime in women in prison

**Assessor Training**

HA, AMcG and VW carried out all participant assessments. HA also carried out assessments on young male and adult female prisoners in two similar studies on head injury in prisoners that overlapped concurrently^1,2^. AMcG and VW were final year clinical psychology trainees. HA, AMcG and VW carried out assessments with the others observing and independently scored/rated responses. Concordance was very high. All were trained/supervised in the use of the assessments by TM. Regular supervision sessions were conducted throughout the study with TM to discuss any queries including in relation to assessment.

^1.^McMillan TM, Aslam H, Crowe E, Seddon E, Barry SJE (2021). Associations between significant head injury and persisting disability and violent crime in women in prison in Scotland, UK: a cross-sectional study. Lancet Psychiatry; 8; 512-20. <https://doi.org/10.1016/S2215-0366(21)00082-1>

^2.^McMillan TM, McVean J, Aslam H, Barry SJE (2023). Associations between significant head injury in male juveniles in prison in Scotland UK and cognitive function, disability and crime: A cross sectional study. PLOS ONE 18(7): e0287312.  <https://doi.org/10.1371/journal.pone.0287312>

**Methods used to Reduce Error in Self-Report**

Table 1

| Variable | Tool | Method | Notes |
| --- | --- | --- | --- |
| Head Injury | OSU-TBI  BISI | Validated interview | Also informing participants about what constitutes a head injury at the start of the interview |
| Disability | Glasgow Outcome  at Discharge Scale | Validated interview | Also utilising information separately from an informant (Personal Prison Officer) |

**Education and Employment History**

Table 2

|  | | | | | |
| --- | --- | --- | --- | --- | --- |
| Variable | Statistic | All (N = 286) | SHI (N = 245) | No-SHI (N = 41) | P-value |
|  |  |  |  |  |  |
| Years of education | N_obs_ (N_miss_) | 284 (2) | 243 (2) | 41 (0) |  |
|  | Median (IQR) | 11 [10, 12] | 11 [10, 12] | 11 [10, 12] |  |
|  | Range | (0, 20) | (0, 20) | (8, 20) | 0.931 |
| School type | N_obs_ (N_miss_) | 284 (2) | 243 (2) | 41 (0) |  |
| None | N (%) | 3 ( 1%) | 3 ( 1%) | 0 ( 0%) |  |
| Mainstream | N (%) | 142 (50%) | 115 (47%) | 27 (66%) |  |
| Mainstream with 1:1 support | N (%) | 44 (15%) | 38 (16%) | 6 (15%) |  |
| Specialist school | N (%) | 95 (33%) | 87 (36%) | 8 (20%) | 0.135 |
| Missed school through truancy | N_obs_ (N_miss_) | 282 (4) | 241 (4) | 41 (0) |  |
| Did not miss school for this reason | N (%) | 35 (12%) | 28 (12%) | 7 (17%) |  |
| Missed school <20 times | N (%) | 48 (17%) | 41 (17%) | 7 (17%) |  |
| Missed school monthly | N (%) | 21 ( 7%) | 20 ( 8%) | 1 ( 2%) |  |
| Missed school weekly | N (%) | 178 (63%) | 152 (63%) | 26 (63%) | 0.509 |
| Missed school through suspension/exclusion | N_obs_ (N_miss_) | 281 (5) | 240 (5) | 41 (0) |  |
| Did not miss school for this reason | N (%) | 65 (23%) | 51 (21%) | 14 (34%) |  |
| Missed school <20 times | N (%) | 161 (57%) | 137 (57%) | 24 (59%) |  |
| Missed school monthly | N (%) | 28 (10%) | 27 (11%) | 1 ( 2%) |  |
| Missed school weekly | N (%) | 27 (10%) | 25 (10%) | 2 ( 5%) | 0.104 |
| Most recent occupation | N_obs_ (N_miss_) | 285 (1) | 244 (1) | 41 (0) |  |
| Unemployed | N (%) | 78 (27%) | 69 (28%) | 9 (22%) |  |
| Elementary | N (%) | 71 (25%) | 62 (25%) | 9 (22%) |  |
| Process plant machine | N (%) | 38 (13%) | 31 (13%) | 7 (17%) |  |
| Sales customer service | N (%) | 10 ( 4%) | 8 ( 3%) | 2 ( 5%) |  |
| Skilled trades, caring, leisure service | N (%) | 67 (24%) | 58 (24%) | 9 (22%) |  |
| Admin and secretarial | N (%) | 1 ( 0%) | 1 ( 0%) | 0 ( 0%) |  |
| Associate professional and technical | N (%) | 6 ( 2%) | 4 ( 2%) | 2 ( 5%) |  |
| Professional | N (%) | 1 ( 0%) | 1 ( 0%) | 0 ( 0%) |  |
| Manager, director and senior | N (%) | 13 ( 5%) | 10 ( 4%) | 3 ( 7%) | 0.645 |
|  | | | | | |

**History of hospital stay with head injury**

Table 3: Self-report of duration of hospital stay with head injury in the SHI group (N 232)

| Duration of admission | N | Percent |
| --- | --- | --- |
| None | 90 | 39 |
| 1-2 | 52 | 22 |
| 3-7 | 42 | 18 |
| 8-14 | 15 | 07 |
| 15-21 | 05 | 02 |
| 22-28 | 03 | 01 |
| 29-56 | 08 | 03 |
| 57-84 | 06 | 03 |
| 85-182 | 07 | 03 |
| >182 | 04 | 02 |

**CNS disorders**

Table 4: Self-report of CNS disorders by group, N (%)

| CNS Disorder | SHI (n 245) | No-SHI (n 41) | All (n 286) |
| --- | --- | --- | --- |
| ADHD | 35 (14) | 1 (2) | 36 (13) |
| Epilepsy/seizures cause unclear | 18 (7) | 3 (6) | 21 (7) |
| Alcohol/drug related seizures | 21 (9) | 2 (4) | 23 (8) |
| Any seizures | 37 (15) | 5 (12) | 42 (15) |
| Learning disability | 12 (5) | 0 | 12 (4) |
| Cerebral anoxia | 8 (2) | 1 (2) | 9 (3) |
| Autistic Spectrum Disorder | 4 (2) | 1 (2) | 5 (2) |
| Stroke | 3 (1) | 1 (2) | 4 (1) |
| Childhood Meningitis | 3 (1) | 3 (6) | 6 (1) |
| Alcohol Related Brain Damage | 2 (1) | 2 (4) | 4 (1) |
| Parkinson’s disease | 1 (<1) | 1 (2) | 2 (<1) |

**Comparison of Trauma Questionnaire Sub-group and total sample**

Table 5: Demographics for participants with and without TLEQ data

|  | | | | | |
| --- | --- | --- | --- | --- | --- |
| Variable | Statistic | All (N = 286) | TLEQ (N = 58) | NoTLEQ (N = 228) | Chisq P-value |
| SHI | N% | 245 (86%) | 48 (83%) | 197 (86%) | 0.619 |
| Age (years) | N_obs_ (N_miss_) | 286 (0) | 58 (0) | 228 (0) |  |
|  | Mean (SD) | 37 (10) | 35 (9) | 37 (11) |  |
|  | Range | (21, 71) | (22, 58) | (21, 71) | 0.105 |
| Ethnicity | N_obs_ (N_miss_) | 285 (1) | 58 (0) | 227 (1) |  |
| White | N (%) | 272 (95%) | 55 (95%) | 217 (96%) |  |
| Non-white | N (%) | 13 ( 5%) | 3 ( 5%) | 10 ( 4%) | 1.000 |
| SIMD high/low | N_obs_ (N_miss_) | 234 (52) | 43 (15) | 191 (37) |  |
| 1:2 (high deprivation) | N (%) | 187 (80%) | 32 (74%) | 155 (81%) |  |
| 3:5 (low deprivation) | N (%) | 47 (20%) | 11 (26%) | 36 (19%) | 0.432 |
| Years of education | N_obs_ (N_miss_) | 284 (2) | 57 (1) | 227 (1) |  |
|  | Median (IQR) | 11 [10, 12] | 10 [8, 12] | 11 [10, 12] |  |
|  | Range | (0, 20) | (0, 17) | (0, 20) | 0.009 |
| School type | N_obs_ (N_miss_) | 284 (2) | 58 (0) | 226 (2) |  |
| None | N (%) | 3 ( 1%) | 1 ( 2%) | 2 ( 1%) |  |
| Mainstream | N (%) | 142 (50%) | 19 (33%) | 123 (54%) |  |
| Mainstream with 1:1 support | N (%) | 44 (15%) | 11 (19%) | 33 (15%) |  |
| Specialist school | N (%) | 95 (33%) | 27 (47%) | 68 (30%) | 0.030 |
| Missed school through truancy | N_obs_ (N_miss_) | 282 (4) | 57 (1) | 225 (3) |  |
| Did not miss school for this reason | N (%) | 35 (12%) | 5 ( 9%) | 30 (13%) |  |
| Missed school <20 times | N (%) | 48 (17%) | 4 ( 7%) | 44 (20%) |  |
| Missed school monthly | N (%) | 21 ( 7%) | 2 ( 4%) | 19 ( 8%) |  |
| Missed school weekly | N (%) | 178 (63%) | 46 (81%) | 132 (59%) | 0.019 |
| Missed school through suspension/exclusion | N_obs_ (N_miss_) | 281 (5) | 56 (2) | 225 (3) |  |
| Did not miss school for this reason | N (%) | 65 (23%) | 10 (18%) | 55 (24%) |  |
| Missed school <20 times | N (%) | 161 (57%) | 35 (62%) | 126 (56%) |  |
| Missed school monthly | N (%) | 28 (10%) | 6 (11%) | 22 (10%) |  |
| Missed school weekly | N (%) | 27 (10%) | 5 ( 9%) | 22 (10%) | 0.742 |
| Most recent occupation | N_obs_ (N_miss_) | 285 (1) | 58 (0) | 227 (1) |  |
| Unemployed | N (%) | 78 (27%) | 24 (41%) | 54 (24%) |  |
| Elementary | N (%) | 71 (25%) | 15 (26%) | 56 (25%) |  |
| Process plant machine | N (%) | 38 (13%) | 7 (12%) | 31 (14%) |  |
| Sales customer service | N (%) | 10 ( 4%) | 1 ( 2%) | 9 ( 4%) |  |
| Skilled trades, caring, leisure service | N (%) | 67 (24%) | 9 (16%) | 58 (26%) |  |
| Admin and secretarial | N (%) | 1 ( 0%) | 0 ( 0%) | 1 ( 0%) |  |
| Associate professional and technical | N (%) | 6 ( 2%) | 1 ( 2%) | 5 ( 2%) |  |
| Professional | N (%) | 1 ( 0%) | 0 ( 0%) | 1 ( 0%) |  |
| Manager, director and senior | N (%) | 13 ( 5%) | 1 ( 2%) | 12 ( 5%) | 0.278 |
|  | | | | | |

**Trauma Measures**

Table 6: History of abuse

|  | | | | | |
| --- | --- | --- | --- | --- | --- |
| Variable | Statistic | All (N = 286) | SHI (N = 245) | No-SHI (N = 41) | Fisher P |
| Any childhood abuse | N_obs_ (N_miss_) | 57 (229) | 48 (197) | 9 (32) |  |
| Yes | N (%) | 37 (65%) | 31 (65%) | 6 (67%) |  |
| No | N (%) | 20 (35%) | 17 (35%) | 3 (33%) | 1.000 |
| Any adult abuse | N_obs_ (N_miss_) | 54 (232) | 47 (198) | 7 (34) |  |
| Yes | N (%) | 41 (76%) | 35 (74%) | 6 (86%) |  |
| No | N (%) | 13 (24%) | 12 (26%) | 1 (14%) | 1.000 |
| Any abuse* | N_obs_ (N_miss_) | 57 (229) | 48 (197) | 9 (32) |  |
| Yes | N (%) | 52 (91%) | 44 (92%) | 8 (89%) |  |
| No | N (%) | 5 ( 9%) | 4 ( 8%) | 1 (11%) | 1.000 |
| PCL5 score | N_obs_ (N_miss_) | 58 (228) | 48 (197) | 10 (31) |  |
|  | Mean (SD) | 36 (18) | 37 (18) | 33 (17) |  |
|  | Range | (0, 80) | (0, 80) | (10, 57) | 0.546 |
| PCL>33 | N_obs_ (N_miss_) | 58 (228) | 48 (197) | 10 (31) |  |
| Yes | N (%) | 34 (59%) | 28 (58%) | 6 (60%) | 1.000 |
| Diagnosis of PTSD | N_obs_ (N_miss_) | 58 (228) | 48 (197) | 10 (31) |  |
| Yes | N (%) | 28 (48%) | 24 (50%) | 4 (40%) | 0.732 |
| Any ACEs | N_obs_ (N_miss_) | 58 (228) | 48 (197) | 10 (31) |  |
| Yes | N (%) | 56 (97%) | 46 (96%) | 10 (100%) | 1.000 |
| Number of ACEs | N_obs_ (N_miss_) | 58 (228) | 48 (197) | 10 (31) |  |
|  | Mean (SD) | 6 (3) | 6 (3) | 5 (3) |  |
|  | Range | (0, 10) | (0, 10) | (1, 10) | 0.548 |
| ACEs>3 | N_obs_ (N_miss_) | 58 (228) | 48 (197) | 10 (31) |  |
| Yes | N (%) | 56 (97%) | 46 (96%) | 10 (100%) | 1.000 |
| Total TLEQ score | N_obs_ (N_miss_) | 58 (228) | 48 (197) | 10 (31) |  |
|  | Mean (SD) | 9 (3) | 9 (3) | 8 (3) |  |
|  | Range | (3, 15) | (3, 15) | (4, 12) | 0.583 |
| *Childhood or adult |  |  |  |  |  |
|  | | | | | |

| Table 7: Trauma history (TLEQ) | | | | |
| --- | --- | --- | --- | --- |
| Variable | Statistic | All (N = 286) | SHI (N = 245) | No.SHI (N = 41) |
| Total TLEQ score | N_obs_ (N_miss_) | 58 (228) | 48 (197) | 10 (31) |
|  | Mean (SD) | 9 (3) | 9 (3) | 8 (3) |
|  | Range | (3, 15) | (3, 15) | (4, 12) |
| Natural disaster | N_obs_ (N_miss_) | 58 (228) | 48 (197) | 10 (31) |
| Yes | N (%) | 7 (12%) | 7 (15%) | 0 ( 0%) |
| Motor vehicle accident | N_obs_ (N_miss_) | 58 (228) | 48 (197) | 10 (31) |
| Yes | N (%) | 26 (45%) | 20 (42%) | 6 (60%) |
| Other accident | N_obs_ (N_miss_) | 58 (228) | 48 (197) | 10 (31) |
| Yes | N (%) | 13 (22%) | 9 (19%) | 4 (40%) |
| Exposure to war | N_obs_ (N_miss_) | 58 (228) | 48 (197) | 10 (31) |
| Yes | N (%) | 0 ( 0%) | 0 ( 0%) | 0 ( 0%) |
| Death of friend/loved one | N_obs_ (N_miss_) | 58 (228) | 48 (197) | 10 (31) |
| Yes | N (%) | 49 (84%) | 41 (85%) | 8 (80%) |
| Loved one accident/assault/illness | N_obs_ (N_miss_) | 58 (228) | 48 (197) | 10 (31) |
| Yes | N (%) | 24 (41%) | 18 (38%) | 6 (60%) |
| Life threatening illness | N_obs_ (N_miss_) | 58 (228) | 48 (197) | 10 (31) |
| Yes | N (%) | 10 (17%) | 8 (17%) | 2 (20%) |
| Robbery with a weapon | N_obs_ (N_miss_) | 58 (228) | 48 (197) | 10 (31) |
| Yes | N (%) | 29 (50%) | 25 (52%) | 4 (40%) |
| Assault by a stranger | N_obs_ (N_miss_) | 58 (228) | 48 (197) | 10 (31) |
| Yes | N (%) | 47 (81%) | 38 (79%) | 9 (90%) |
| Witness assault | N_obs_ (N_miss_) | 58 (228) | 48 (197) | 10 (31) |
| Yes | N (%) | 46 (79%) | 40 (83%) | 6 (60%) |
| Threat of serious harm | N_obs_ (N_miss_) | 58 (228) | 48 (197) | 10 (31) |
| Yes | N (%) | 39 (67%) | 34 (71%) | 5 (50%) |
| Physical child abuse | N_obs_ (N_miss_) | 56 (230) | 47 (198) | 9 (32) |
| No | N (%) | 24 (43%) | 21 (45%) | 3 (33%) |
| 1-2 times | N (%) | 1 ( 2%) | 1 ( 2%) | 0 ( 0%) |
| 3+ times | N (%) | 31 (55%) | 25 (53%) | 6 (67%) |
| Fear of parental violence | N_obs_ (N_miss_) | 33 (253) | 27 (218) | 6 (35) |
| Yes | N (%) | 27 (82%) | 22 (81%) | 5 (83%) |
| Witnessed family violence | N_obs_ (N_miss_) | 58 (228) | 48 (197) | 10 (31) |
| Yes | N (%) | 35 (60%) | 30 (62%) | 5 (50%) |
| Partner violence | N_obs_ (N_miss_) | 54 (232) | 46 (199) | 8 (33) |
| No | N (%) | 13 (24%) | 12 (26%) | 1 (12%) |
| 1-2 times | N (%) | 10 (19%) | 9 (20%) | 1 (12%) |
| 3+ times | N (%) | 31 (57%) | 25 (54%) | 6 (75%) |
| Fear of partner violence | N_obs_ (N_miss_) | 41 (245) | 34 (211) | 7 (34) |
| Yes | N (%) | 15 (37%) | 14 (41%) | 1 (14%) |
| Childhood sexual abuse | N_obs_ (N_miss_) | 52 (234) | 45 (200) | 7 (34) |
| No | N (%) | 40 (77%) | 34 (76%) | 6 (86%) |
| 1-2 times | N (%) | 4 ( 8%) | 3 ( 7%) | 1 (14%) |
| 3+ times | N (%) | 8 (15%) | 8 (18%) | 0 ( 0%) |
| Fear of childhood sexual abuse | N_obs_ (N_miss_) | 12 (274) | 11 (234) | 1 (40) |
| Yes | N (%) | 9 (75%) | 8 (73%) | 1 (100%) |
| Adult sexual abuse | N_obs_ (N_miss_) | 52 (234) | 45 (200) | 7 (34) |
| No | N (%) | 50 (96%) | 43 (96%) | 7 (100%) |
| 1-2 times | N (%) | 1 ( 2%) | 1 ( 2%) | 0 ( 0%) |
| 3+ times | N (%) | 1 ( 2%) | 1 ( 2%) | 0 ( 0%) |
| Fear of adult sexual abuse | N_obs_ (N_miss_) | 4 (282) | 4 (241) | 0 (41) |
| Yes | N (%) | 1 (25%) | 1 (25%) | 0 (-) |
| Unwanted sexual attention | N_obs_ (N_miss_) | 58 (228) | 48 (197) | 10 (31) |
| Yes | N (%) | 7 (12%) | 6 (12%) | 1 (10%) |
| Stalking | N_obs_ (N_miss_) | 58 (228) | 48 (197) | 10 (31) |
| Yes | N (%) | 9 (16%) | 8 (17%) | 1 (10%) |
| Miscarriage | N_obs_ (N_miss_) | 58 (228) | 48 (197) | 10 (31) |
| Yes | N (%) | 24 (41%) | 20 (42%) | 4 (40%) |
| Abortion | N_obs_ (N_miss_) | 58 (228) | 48 (197) | 10 (31) |
| Yes | N (%) | 10 (17%) | 8 (17%) | 2 (20%) |
| Interpersonal trauma | N_obs_ (N_miss_) | 58 (228) | 48 (197) | 10 (31) |
| Yes | N (%) | 44 (76%) | 37 (77%) | 7 (70%) |
| Other | N_obs_ (N_miss_) | 58 (228) | 48 (197) | 10 (31) |
| Yes | N (%) | 22 (38%) | 18 (38%) | 4 (40%) |
|  | | | | |

**Disability**

Table 8

|  | | | | | |
| --- | --- | --- | --- | --- | --- |
| Variable | Statistic | All (N = 286) | SHI (N = 245) | No.SHI (N = 41) | Chisq P |
| GODS: any cause disability | N_obs_ (N_miss_) | 286 (0) | 245 (0) | 41 (0) |  |
| No disability | N (%) | 122 (43%) | 99 (40%) | 23 (56%) |  |
| Disability | N (%) | 164 (57%) | 146 (60%) | 18 (44%) | 0.087 |
| GODS: other cause disability categories | N_obs_ (N_miss_) | 286 (0) | 245 (0) | 41 (0) |  |
| Good recovery | N (%) | 152 (53%) | 129 (53%) | 23 (56%) |  |
| Moderate disability | N (%) | 102 (36%) | 89 (36%) | 13 (32%) |  |
| Severe disability | N (%) | 32 (11%) | 27 (11%) | 5 (12%) | 0.847 |
|  | | | | | |

**Analysis of Cognitive Test Scores**

The cognitive test z-scores were adjusted for age, years of education and delayed word memory score. This was done by fitting a linear model to the raw cognitive test scores, adjusting for the aforementioned covariates, extracting the residuals and standardising them to mean 0 and SD 1. The overall cognitive impairment z-score was calculated as a mean of the positive z-scores for Symbol Digit, List Learning and the negative Trails Part B z-time, resulting in a z-score for which lower values represent greater cognitive impairment. If an individual score was missing, then the overall score was calculated as the mean of the available scores (see table 9 and figures below).

Table 9: Outcomes: Cognitive impairment

(z-scores are adjusted for age, years of education and delayed word memory score)

|  | | | | | |
| --- | --- | --- | --- | --- | --- |
| Variable | Statistic | All (N = 286) | SHI (N = 245) | No-SHI (N = 41) | P-value |
| Word memory immediate score | N_obs_ (N_miss_) | 284 (2) | 243 (2) | 41 (0) |  |
|  | Mean (SD) | 34.0 (4.1) | 33.9 (4.1) | 34.1 (4.0) |  |
|  | Range | (21.0, 50.0) | (21.0, 50.0) | (24.0, 39.0) | 0.774 |
| Word memory delayed score | N_obs_ (N_miss_) | 284 (2) | 243 (2) | 41 (0) |  |
|  | Mean (SD) | 34.8 (4.2) | 34.8 (4.3) | 35.1 (3.6) |  |
|  | Range | (19.0, 40.0) | (19.0, 40.0) | (24.0, 39.0) | 0.686 |
| Word memory consistency score | N_obs_ (N_miss_) | 266 (20) | 228 (17) | 38 (3) |  |
|  | Mean (SD) | 33.2 (4.2) | 33.1 (4.3) | 33.9 (3.0) |  |
|  | Range | (9.0, 40.0) | (9.0, 40.0) | (25.0, 38.0) | 0.254 |
| Symbol digit score | N_obs_ (N_miss_) | 278 (8) | 237 (8) | 41 (0) |  |
|  | Mean (SD) | 42.4 (10.4) | 42.4 (10.5) | 42.7 (9.5) |  |
|  | Range | (16.0, 70.0) | (16.0, 70.0) | (20.0, 59.0) | 0.865 |
| Symbol digit adjusted z-score | N_obs_ (N_miss_) | 276 (10) | 235 (10) | 41 (0) |  |
|  | Mean (SD) | 0.000 (1.000) | -0.001 (1.024) | 0.003 (0.861) |  |
|  | Range | (-2.452, 2.983) | (-2.452, 2.983) | (-1.800, 1.484) | 0.981 |
| AMIPB (list learning) score | N_obs_ (N_miss_) | 278 (8) | 237 (8) | 41 (0) |  |
|  | Mean (SD) | 36.4 (9.0) | 36.2 (9.0) | 37.6 (9.0) |  |
|  | Range | (15.0, 61.0) | (15.0, 61.0) | (20.0, 57.0) | 0.332 |
| AMIPB (list learning) adjusted z-score | N_obs_ (N_miss_) | 276 (10) | 235 (10) | 41 (0) |  |
|  | Mean (SD) | 0.000 (1.000) | -0.021 (1.004) | 0.118 (0.979) |  |
|  | Range | (-2.281, 2.658) | (-2.281, 2.658) | (-2.046, 1.962) | 0.412 |
| Trail Making Test part B | N_obs_ (N_miss_) | 267 (19) | 228 (17) | 39 (2) |  |
|  | Mean (SD) | 90.9 (41.1) | 91.8 (42.2) | 86.0 (34.0) |  |
|  | Range | (2.2, 252.0) | (2.2, 252.0) | (46.0, 211.0) | 0.416 |
| Trail Making Test part B adjusted z | N_obs_ (N_miss_) | 265 (21) | 226 (19) | 39 (2) |  |
|  | Mean (SD) | 0.000 (1.000) | 0.020 (1.031) | -0.115 (0.797) |  |
|  | Range | (-2.014, 4.090) | (-2.014, 4.090) | (-1.376, 2.922) | 0.437 |
| Overall cognitive impairment (adjusted z-score)* | N_obs_ (N_miss_) | 281 (5) | 240 (5) | 41 (0) |  |
|  | Mean (SD) | 0.000 (1.000) | -0.019 (1.025) | 0.112 (0.839) |  |
|  | Range | (-3.467, 2.785) | (-3.467, 2.785) | (-1.602, 1.366) | 0.441 |
| Overall cognitive impairment (adjusted z-score)* for those with word memory delayed>33 | N_obs_ (N_miss_) | 203 (2) | 171 (2) | 32 (0) |  |
|  | Mean (SD) | 0.033 (0.980) | 0.012 (0.999) | 0.147 (0.879) |  |
|  | Range | (-2.739, 2.629) | (-2.739, 2.629) | (-1.602, 1.366) | 0.476 |
| * For overall cognitive impairment, a lower score represents greater cognitive impairment |  |  |  |  |  |
|  | | | | | |

Figures 1: Raw and adjusted cognitive test scores by group


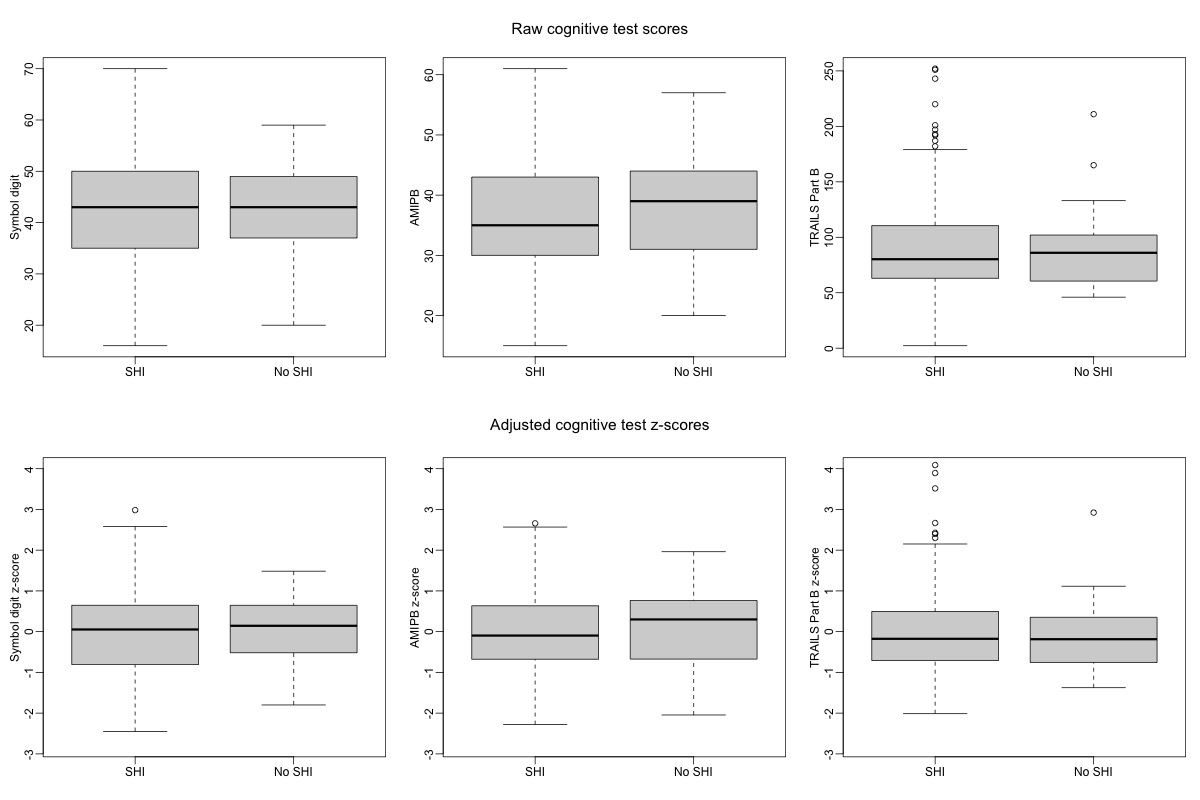


Figures 2: Overall cognitive impairment by group (z-scores)


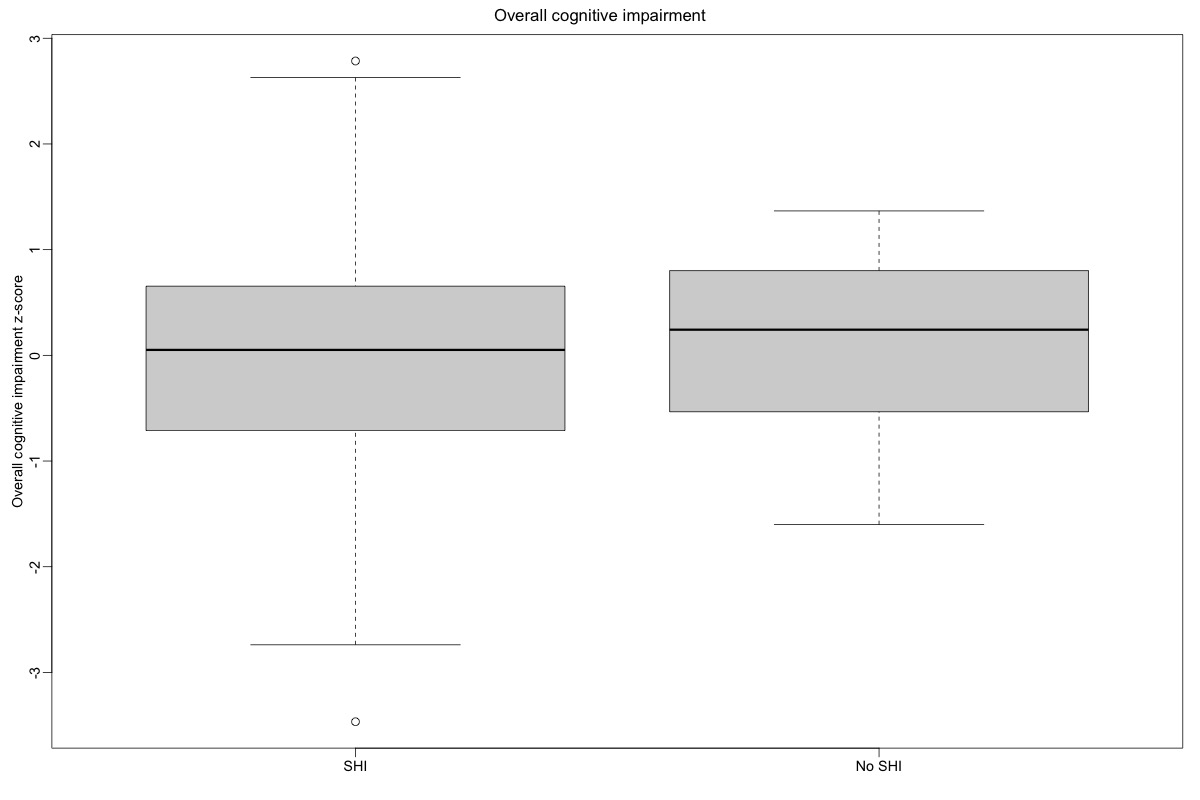


**Comparison of Cognitive Test Scores with Test Norms**

Published norms for the general population, stratified where available for age, education and gender, were used to create z-scores for each individual and from these mean deviations from the norms are presented.

Table 10: Comparisons between cognitive test scores and published test norms

| Test | Stratification of  Test Norm | Test Norm (Mean; SD) | Males in prison (N=286); Mean  Z-score difference | P value |
| --- | --- | --- | --- | --- |
| Symbol Digit Modalities Test^1^ | Age, education, gender | 45.6; 12.0 | 0.27 | .3936 |
| Auditory Verbal Learning Test^2^ | Age | 50.3; 9.7 | 1.43 | .0764 |
| Trail Making Test B^3^ | Age | 58.5; 16.4 | -1.98 | .0239 |

1. Kiely KM, Butterworth P, Watson N et al (2014). The Symbol Digit Modalities Test: Normative Data from a Large Nationally Representative Sample of Australians. Archives of Clinical Neuropsychology, 29; 767–775
2. Coughlan AK & Hollows SE. The Adult Memory and Information Processing Battery Test Manual. Psychology Department, University of Leeds, Leeds, UK 1985
3. Tombaugh T. Trail Making Test A and B: Normative data stratified by age and education. Archives of Clin Neuropsychol 2004: 19:203-214

**Multivariate Models for Outcome Variables**

Table 11: Model estimates and 95% confidence intervals

|  | | |
| --- | --- | --- |
| Variable | Univariable* | Multivariable* |
| SHI disability |  |  |
| Clinical depression | - | 2.07 (1.03,4.16) |
| Clinical anxiety | - | 1.92 (1.06,3.46) |
| Self-reported problematic alcohol or drug use | - | 3.64 (1.34,9.89) |
| CNS diagnosis | - | 1.45 (0.81,2.61) |
| Any cause disability |  |  |
| SHI | 1.88 (0.97,3.67) | 1.15 (0.54,2.43) |
| Clinical depression |  | 1.47 (0.63,3.43) |
| Clinical anxiety |  | 4.67 (2.54,8.58) |
| Self-reported problematic alcohol or drug use |  | 3.62 (1.72,7.61) |
| CNS diagnosis |  | 2.52 (1.37,4.66) |
| Violent offences |  |  |
| SHI | 3.31 (1.51,7.24) | 2.94 (1.25,6.89) |
| Clinical depression |  | 0.78 (0.23,2.63) |
| Clinical anxiety |  | 2.44 (0.99,6.02) |
| Self-reported problematic alcohol or drug use |  | 4.21 (1.91,9.24) |
| CNS diagnosis |  | 1.28 (0.53,3.07) |
| Number of convictions |  |  |
| SHI | 1.96 (0.61,6.29) | 1.93 (0.65,5.77) |
| Clinical depression |  | 1.33 (0.64,2.77) |
| Clinical anxiety |  | 0.79 (0.42,1.51) |
| Self-reported problematic alcohol or drug use |  | 5.04 (1.13,22.46) |
| CNS diagnosis |  | 1.05 (0.57,1.94) |
| Number of convictions (truncated to 100) |  |  |
| SHI | 1.59 (1.00,2.53) | 1.41 (0.92,2.16) |
| Clinical depression |  | 1.39 (1.03,1.88) |
| Clinical anxiety |  | 1.02 (0.78,1.34) |
| Self-reported problematic alcohol or drug use |  | 3.82 (2.14,6.82) |
| CNS diagnosis |  | 1.22 (0.94,1.57) |
| Longest sentence |  |  |
| SHI | 0.83 (0.58,1.18) | 0.78 (0.54,1.11) |
| Clinical depression |  | 1.32 (0.92,1.88) |
| Clinical anxiety |  | 1.06 (0.79,1.42) |
| Self-reported problematic alcohol or drug use |  | 1.07 (0.74,1.53) |
| CNS diagnosis |  | 0.88 (0.65,1.17) |
| Cognitive impairment |  |  |
| SHI | -0.13 (-0.46,0.20) | -0.04 (-0.37,0.30) |
| Clinical depression |  | -0.05 (-0.38,0.27) |
| Clinical anxiety |  | -0.11 (-0.37,0.14) |
| Self-reported problematic alcohol or drug use |  | -0.23 (-0.54,0.08) |
| CNS diagnosis |  | -0.26 (-0.51,-0.01) |
| *ORs for disability/violent offences; RRs for convictions/sentence; Mean difference for cognitive |  |  |
|  | | |

**Disability Outcome**

Figure 3: Disability associated with SHI


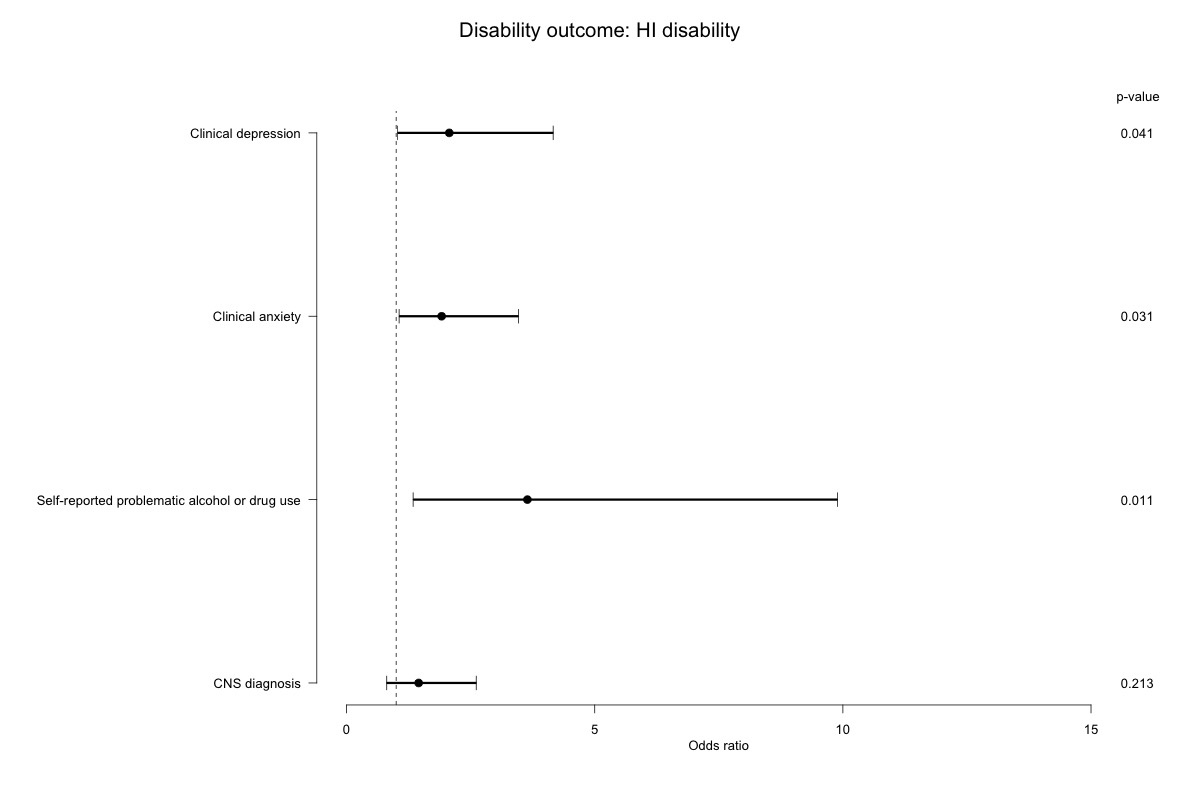


Figure 4: Disability from any cause


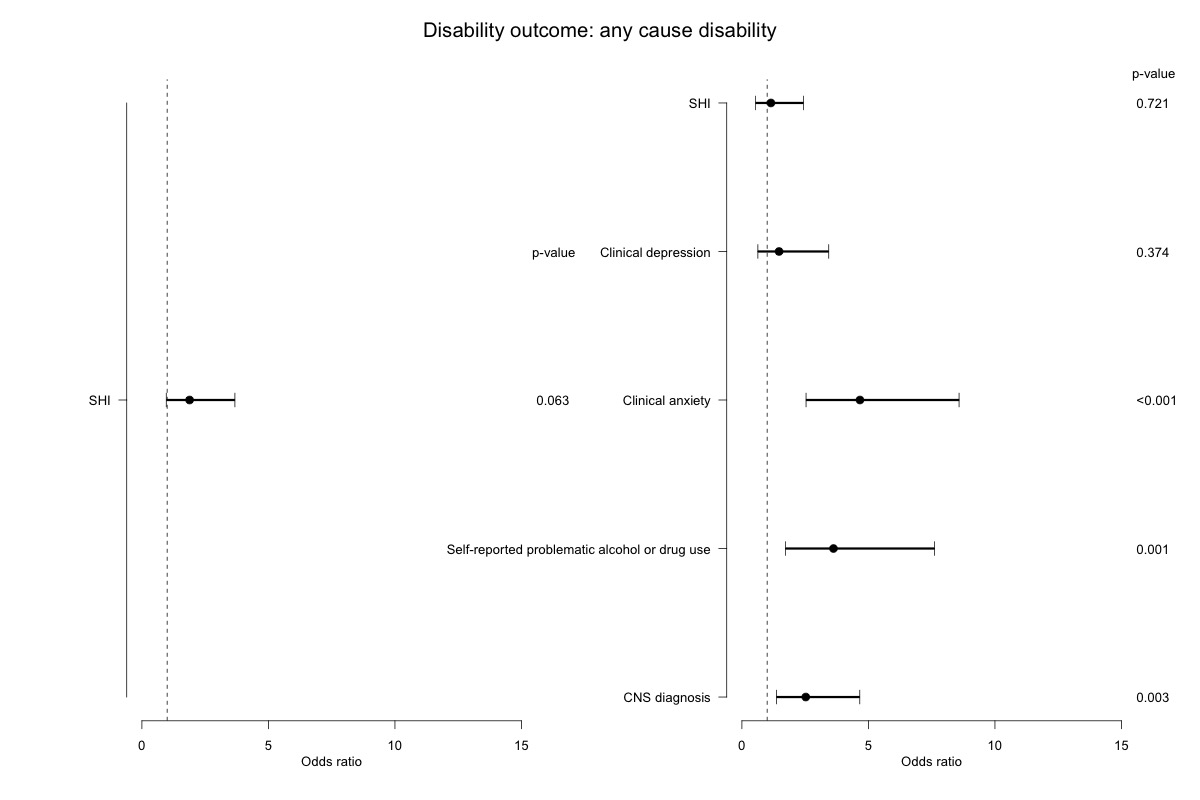


**Cognitive outcome**

Figure 5: Cognitive outcome (z-score)


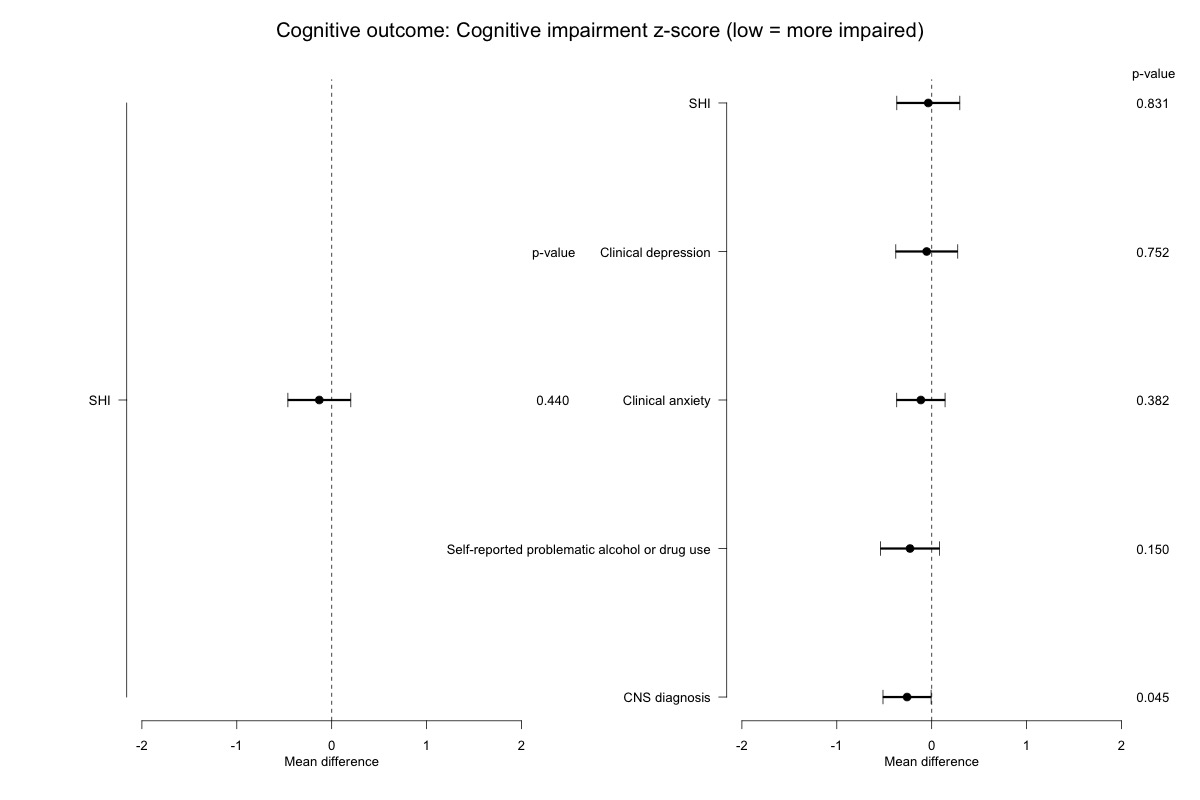


**Offending Outcome**

Figure 6: Longest prison sentence


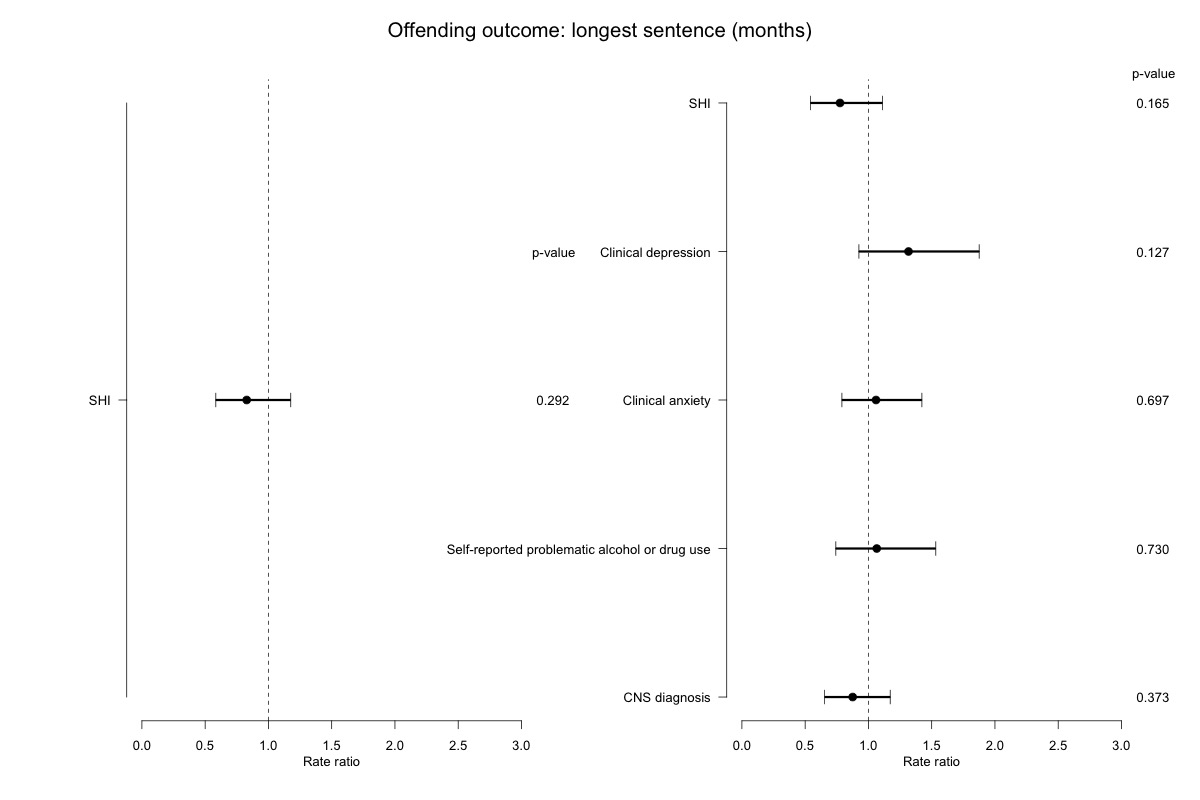


Figure 7: Number of convictions


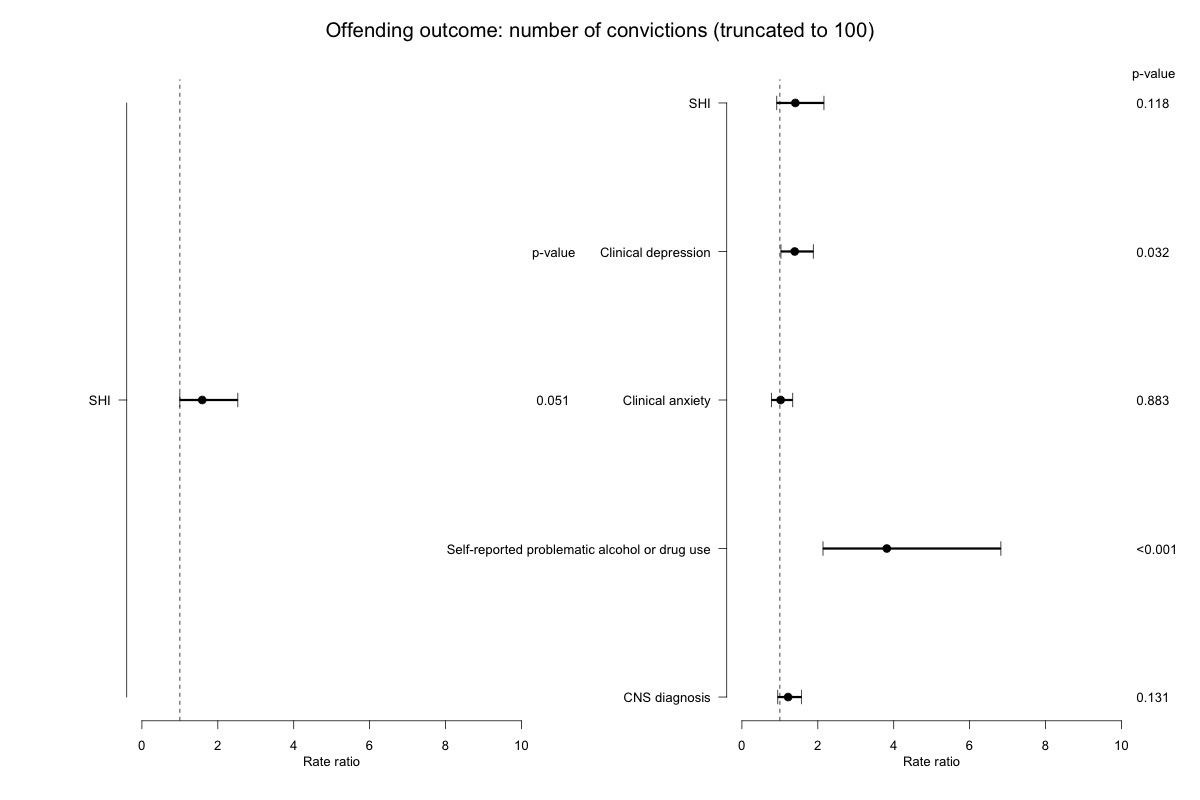


Figure 8: Violent offences

**
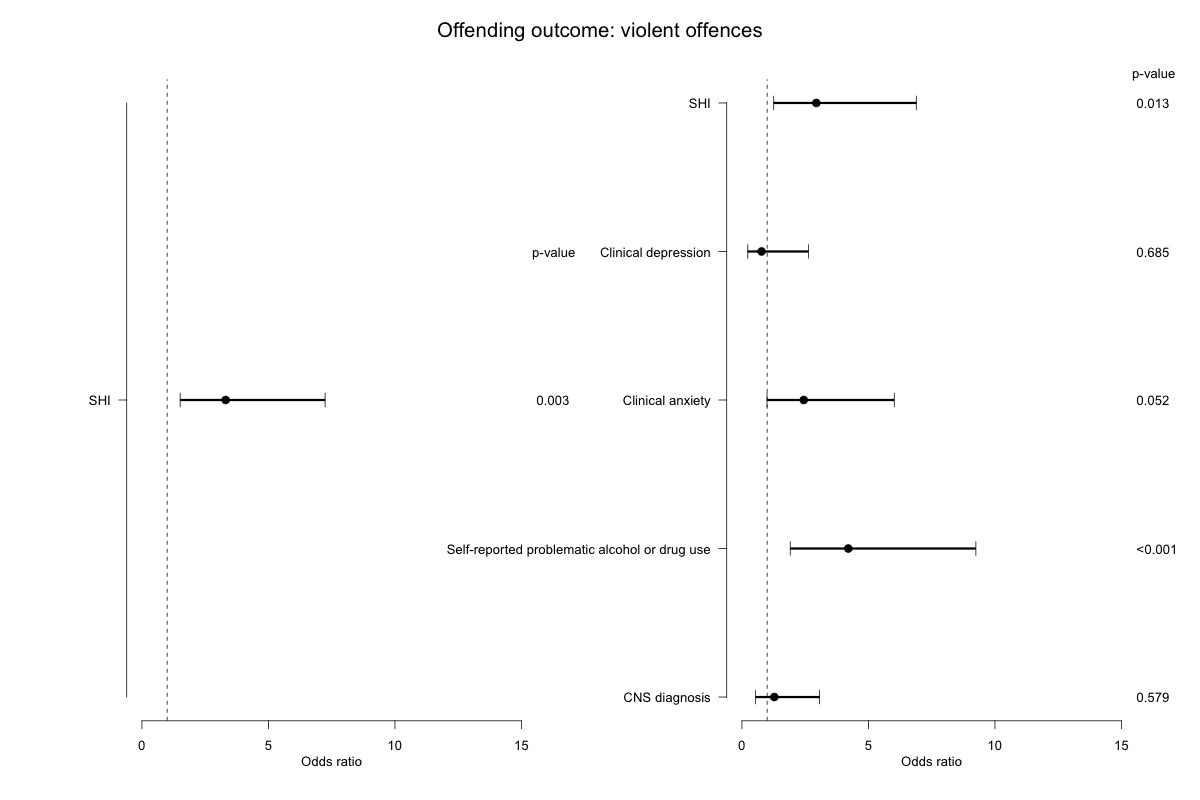
**
